# Coffee

**Published:** 1858-09

**Authors:** 


### COFFEE.

UNTIL Chemistry has a better claim than it has now, to be called one of the exact sciences, we had better give its *dicta* a wide berth as to their practical application in reference to food and drink.

When a concentrated miasm is mingled with the air a man breathes, he will in a few hours sicken and die; but miasm is of such an intangible, aerial nature, that chemistry has no test sufficiently delicate to detect its presence. And if, because chemistry can find no poison there, a man persists in breathing it, he will most certainly suffer the gravest consequences.

The solidified oil of roses, with the delightful fragrance of which we are so familiar as it comes in its natural state from the opening bud, contains precisely the same elements, and in the same proportions, as the gas which lights our streets, and churches, and houses of abode; that is to say, chemistry detects nothing else; and yet we know, from the diversity of odor, there must be something in coal-gas or crystallized rose-oil for which the chemist, as yet, has no test.

Several chemists who have had a reputation, and have one still, have certified that they have found no material difference between the milk of farm-house cows and those fed mainly on distillery swill, and confined to filthy apartments. It is true, they do not tell us by what process they arrived at these conclusions; but the bare fact of their finding no deleterious substances in the milk of cabined, confined, swill-fed milch-cows, does not prove that no bad quality existed, but simply that they were not able to detect it; while we all know, that just as certainly will infants sicken and die who are fed on swill-milk, as men will sicken and die who breathe a miasmatic atmosphere. So that, in practical cases like these, the masses must be guided in their habits by their observation and their common-sense, and let the vagaries of science and scientific men go out to browse and mature, or, in common phrase, "go to grass."

Some say that coffee is poisonous, because it has strychnine in it. Suppose it is granted that there is strychnine in coffee, and that a single grain of strychnine is so poisonous as to destroy life in a few moments, that does not prove that strychnine in coffee is poisonous; because there may be an element in the coffee which would not only nullify the poisonous quality, but render it absolutely safe, healthful, and nutritious.

All the *elements* of strychnine are found in coffee, but that strychnine, as such, should exist in coffee, is an absurdity: if it did, without a counteracting constituent, we should soon die from its daily use; when, in fact, we do not die, as a nation, but absolutely, as a nation, grow longer-lived, either on coffee, or in spite of it—and if the latter, it cannot be very hurtful.

The air we breathe has the same elements as aqua fortis, but in different proportion, and that makes all the difference in the world. Coffee and strychnine are composed, as far as chemistry can inform us, of nitrogen, oxygen, hydrogen, and carbon. But,

| | | | | | | | | |
|---|---|---|---|---|---|---|---|---|
| Coffee contains of nitrogen, | 2 parts; | oxygen, | 2; | hydrogen, | 5; | carbon, | 8. |
| Strychnine | " " | 2 " | " | 4 | " | 23 | " | 44. |

To show, in familiar substances, what difference is made in the quality of an article, simply by a difference in the proportion of the chemical constituents, we may state those of the air we breathe and aqua fortis or nitric acid:

| | | | | |
|---|---|---|---|---|
| The air contains, by weight, in round numbers, oxygen, | 25; | nitrogen, | 75. |
| Aqua fortis, " " | " | 40; | " | 14. |

So that any article of food or drink may have in it the elements of the most deadly poison, and yet, in consequence of a difference in the proportions of those elements, it may be not merely innocuous, but very healthful, and highly nutritious. The practical result of the whole matter is simply this: it is immaterial what coffee contains, as long as its immediate effects are agreeable; while, as to its ultimate effects, a man or woman may use it safely to the age of three score years and ten. If there are some persons whom it affects unpleasantly, the dictate of common sense is for them to let it alone, at least for the present. Such persons have brought themselves to this condition of things by their beastly use of the beverage, and turn round to kick the stone they stubbed their foot against. Being better photographed in the fable of a fox without a tail, or that of the dog in the manger, we dismiss the subject.

Thus it is that men of confined views, of limited observation, and still less information, assert with cool confidence that coffee is poisonous—just as if they knew all of anything, when really they know all of nothing. The truly learned are cautious in their declarations; they fear to deal in other than general expressions of opinion, and leave a bridge of retreat. They are the last men in the world to use sweeping adjectives: "certain," and "always," and "never," with words like them, are not found in their modest vocabulary; "it appears," "it seems," "it is probable," are frequent phrases in their conversations and writings.

As to the bold assertions that coffee is not healthful, is not nutritious, is poisonous, we must appeal to our general observation. People use it daily, and yet live to threescore and ten. For a hundred years past, it is more and more used by all who speak the English language; and yet, within the last hundred years, the average of civilized life is greater, by several years, and the Anglo-Saxon races have increased more rapidly than in any other age. To say that they have done this, in spite of the increasing use of coffee, and that its ill effects will begin to be felt before a great while, is nothing more than the impudent assertion of a cornered ignoramus.

A single fact sometimes demonstrates a great truth. Within three years, a party bringing the mails from the Rocky Mountains were overtaken by a snow-storm; and in their official report, they stated, that for two weeks their *entire* subsistence was a few bags of coffee, on which they travelled. Had there been no nutriment in the coffee, they must have died. To this, the anti-coffee man will reply: "We don't know that"—nor do they know anything else. Meanwhile, if some persons will not use coffee, there will be more left for those who do; and as for ourselves, we ask the liberty of being allowed to eat and drink what we like; and we do most cordially allow that liberty to others, and "no questions asked."

Minute chemical analysis says, that the essence of coffee and tea are identical. We believe that both are nutritious and healthful, when taken, with one restriction, as a beverage—never increase it in frequency, strength, or quantity.

M. Caron, of Paris, says, that minute investigation seems to justify his assertion, that the nutritious properties of the French beverage of coffee and milk, half and half, are neutralized by the astringent qualities of the coffee, for coffee and milk in a bottle did not begin to decompose for twenty-seven days; but milk and sugar began to decompose in three days; he infers, therefore, that coffee interferes with the digestion of the milk; and that, therefore, most of the nervous and allied disorders, such as neuralgias, irritations, and hysteria, which affect those who live in cities, may be very properly attributed to the coffee they use. It is just such refinements as these which fill the world with so much nonsense. Putting milk and coffee into a bottle, is not putting it into a human stomach. Who can say that it is not the milk which hurts the coffee? Besides, all the milk or cream generally used by Americans in coffee, would not amount to a quart in a week, consequently, if the coffee did retard its digestion, it could be no very great affair.

It is just the same refining nonsense which induced some man to publish a book, a few years ago, in New York, the burden of which was, that we used too much bread, that it made the bones brittle, that if we continued to use it so freely, a man would fall and break all to pieces, the first time he stumped his toe; closing with the assertion, that the great remedy for all this was to live mainly on grapes, any quantity of which, his friend Dr. —— was prepared to furnish. "In the name of the prophet — FIGS!!" The general sense of observant persons is, that the proper preparation and moderate use of coffee is, considering our general habits of life, nutritious, safe, and healthful. As to its rational employment, we have already remarked: it should be roasted and ground immediately previous to its use, not burned in an open pan, but just browned in a closed vessel, and boiled in such a way as to prevent the loss of any of its essential qualities—a simple and perfect device for which is found in what is now generally known as "The Old Dominion Coffee Pot," manufactured by ARTHUR, BURNHAM & GILROY, of Philadelphia, Pa.

It may be instructive to the mental philosopher, to report an incident in our office the other day, as illustrative of the extreme pugnacity of even cultivated minds, as to some things that are new, contrasted with their shark-like voracity in swallowing others. A professional reader of our JOURNAL called to make inquiry as to the coffee-pot we had mentioned in a previous number, and insisted that we must be mistaken as to any advantage of boiling it two hours; that he had often prepared his own coffee, and always found that it became bitter, if boiled even half an hour. We suggested, that perhaps he boiled all the good qualities away, and retained the refuse. "But," said he, "if you confine the steam, it will explode." "Precisely," we rejoined, "as your retort explodes when you distil alcohol or water!" For we knew he had a laboratory of his own. He determined to buy an "Old Dominion" forthwith. There are not a few who estimate the measure of their own wit and wisdom by the number of objections they can raise against what is claimed to be new and useful. In their inordinate fear of being imposed upon, they run to the other extreme: forgetting that quite as great a wrong is done to themselves and to society in opposing what is really good, as in blindly advocating a mere pretence.

